# Endovascular Repair of an Inflammatory Abdominal Aortic Aneurysm Combined with a Congenital Pelvic Kidney: Case Report and Literature Review

**DOI:** 10.1055/s-0042-1748961

**Published:** 2022-11-01

**Authors:** Spyros Papadoulas, Natasa Kouri, Andreas Tsimpoukis, Petros Zampakis, Marios Papasotiriou, Konstantinos G. Moulakakis, Stavros K. Kakkos

**Affiliations:** 1 Department of Vascular Surgery, University Hospital of Patras, Patras, Greece; 2 Departement of Radiology, University Hospital of Patras, Patras, Greece; 3 Department of Nephrology, University Hospital of Patras, Patras, Greece

**Keywords:** inflammatory, abdominal aortic aneurysm, congenital pelvic kidney, endovascular aneurysm repair

## Abstract

The coexistence of an abdominal aortic aneurysm and a congenital pelvic kidney is extremely rare. We present a 66-year-old male with an inflammatory aneurysm and an aberrant origin of the superior mesenteric artery. The inflammatory infrarenal abdominal aortic aneurysm with a congenital left pelvic kidney was successfully treated with endovascular repair. Coverage of one out of the two renal ectopic arteries was performed, without clinical evidence of renal function impairment.

## Introduction

Congenital pelvic kidney (CPK) is a congenital anomaly which occurs due to failure of the embryological kidney to ascend normally and reside in the lumbar retroperitoneal space. Pelvic kidney occurs during the 6th to 9th week of gestation and is present in 1 out of 2,100 to 3,000 births. 1

The coexistence of an abdominal aortic aneurysm (AAA) and a CPK is rare, with less than 50 cases reported in the literature. 2 Herein, we report a 66-year-old male with an inflammatory AAA (IAAA) and a coexistent CPK. We were not able to find a similar case with an IAAA in the literature. We have also reviewed the literature focusing especially on the cases of AAA and CPK. We discuss and analyze the treatment options to repair the AAA while preserving the CPK. 2 3 4

## Case Presentation

**Fig. 1:**
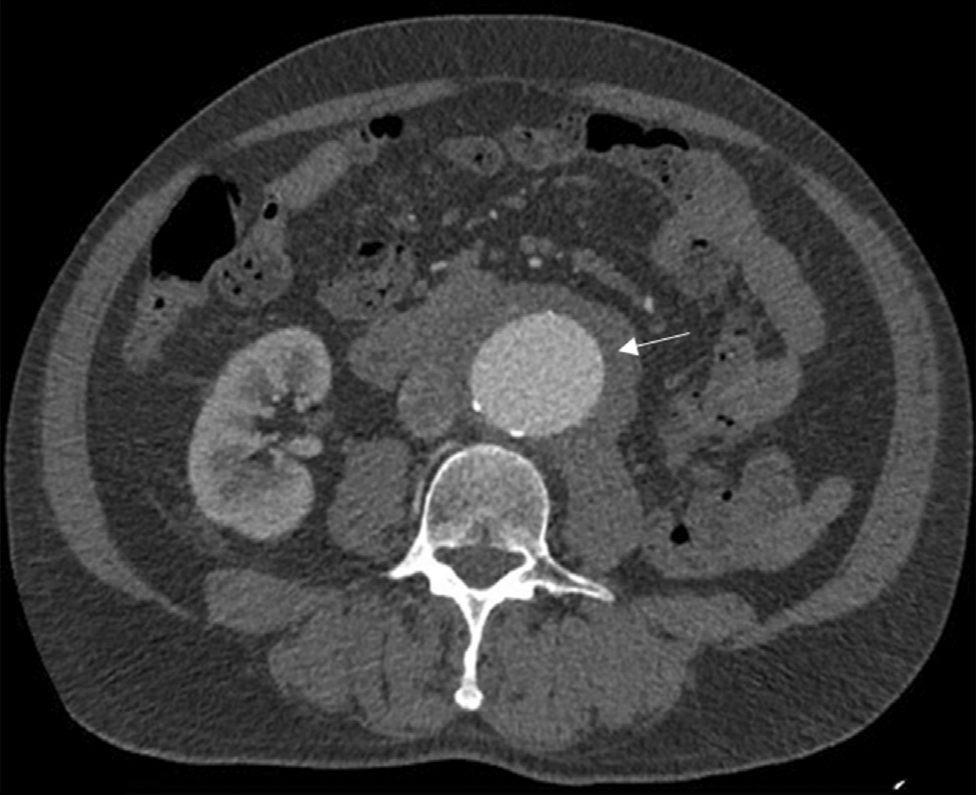
The inflammatory aneurysm.

**Fig. 2:**
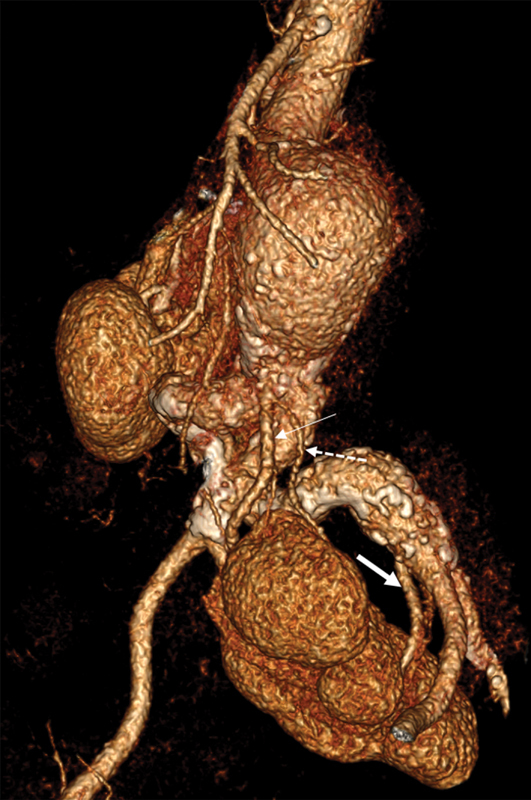
Preoperative computed tomography angiography depicting the left congenital pelvic kidney and the two aberrant renal arteries (RAs) (thick arrow: distal RA, thin arrow: proximal RA, dotted arrow: small branch of the proximal RA).

**Fig. 3:**
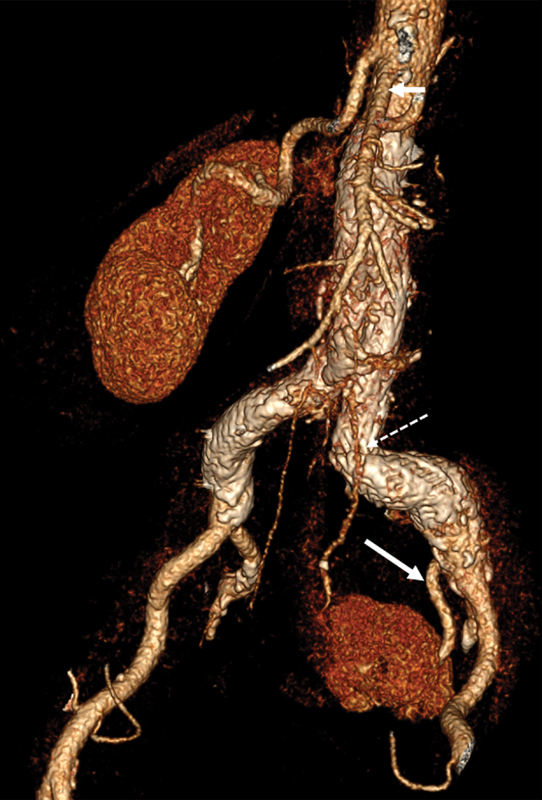
Three-month postoperative computed tomography angiography depicting the normal flow in the distal left renal artery (LRA; solid arrow). The proximal LRA is slightly opacified, probably via antegrade flow from the Type IIb endoleak (dotted arrow).

A 66-year-old male presented with an asymptomatic AAA. On physical examination, a pulsatile abdominal mass was palpated, and pulses were present in both lower extremities. Past medical history included smoking, hypertension, hyperlipidemia, and moderate carotid artery stenosis. Surgical history included an inguinal hernia repair 2 years ago. Serum creatinine concentration was normal. A computed tomography angiography (CTA) revealed a 5.8-cm fusiform infrarenal IAAA surrounded by a perianeurysmal inflammatory tissue 1.5cm thick ( Fig. 1 ). The right kidney was normally positioned. A normal sized CPK with aberrant arterial supply was apparent in the pelvis. The left renal vasculature included one proximal artery originating from the inferior infrarenal abdominal aorta just proximal to the aortic bifurcation (4.5 mm in diameter) and a distal one, arising from the left common iliac bifurcation (4.5 mm in diameter; Fig. 2 ). Furthermore, the superior mesenteric artery (SMA) originated just caudal to the right renal artery (RRA; Fig. 3 ). Both common iliac arteries presented with aneurysmal dilatation and mild tortuosity. Radioisotope renography with Tc-99m-DTPA combined with dimercaptosuccinic acid showed a uniform contribution (50%) of both kidneys to renal function.

As an open reconstruction was deemed risky due to the dense inflammation around the aneurysm, a hybrid procedure was proposed to the patient, consisting of an open bypass (between the external iliac and the proximal renal artery with a saphenous vein graft) and an endovascular aneurysm repair (EVAR). 1 The patient declined the open or hybrid repair options. Therefore, we proceeded with EVAR, preserving the distal left renal artery (LRA), and sacrificing the proximal one.

A standard EVAR procedure with a Gore C3 Excluder AAA endoprothesis (26 mm × 14 mm × 16 mm, W.L. Gore & Associates, Newark, DE) was performed under general orotracheal anesthesia, through bilateral common femoral cut-down access. Digital subtraction angiography (DSA) with an oblique view was performed to determine the exact position of the orifice of the SMA. Subsequently, DSA with a frontal view located the origin of the RRA. Proximal initial low apposition of the main stent graft was corrected with an aortic cuff (Gore 28.5 mm × 33 mm). Completion DSA demonstrated complete aneurysm exclusion and normal patency of the SMA, RRA, and distal LRA. The proximal LRA was not opacified, as its orifice was intentionally covered by the graft. Total contrast volume was 260mL and dose area product was 138mGY/3.95MgYm 2 .

The patient had an uneventful recovery and was discharged on the second postoperative day with a daily aspirin (100mg) and simvastatin. A 3-month postoperative CTA revealed normal stent-graft fixation and a minor Type IIb endoleak from lumbar arteries. Although approximately 30 to 40% of the pelvic kidney was infarcted due to the coverage of the accessory renal artery, the renal function remained normal ( Fig. 3 ). Blood pressure and serum creatinine remained stable postoperation (preoperative serum creatinine: 1.6 mg/dL and postoperative serum creatinine: 1.4 mg/dL).

## Discussion

Pelvic is the rarest of the six types of renal ectopias. The remaining five are the lumbar, abdominal, cephalad, thoracic, and crossed ectopia. 5 Fusion phenomena may take place between two ectopic pelvic kidneys forming a solitary congenital fused pelvic kidney (CFPK, the so-called “cake” kidney). 6 CPK and CFPK are associated with aberrant arterial supply originating from different levels of the aorta, the bifurcation, and the iliac arteries. 2

Other renal malpositioning configurations include the horseshoe kidney and the crossed fused renal ectopia (CFRE). In two out of six types of CFRE, the fused kidney differs from the CFPK because it lies unilaterally in the lumbar or iliac fossa and the ureter crosses the midline (“lump” or “disc” kidney).

IAAA have a thickened aortic wall and account for 5% of all aortic aneurysms. Open repair presents distinct challenges due to the dense adhesions and adherence of adjacent structures to the aorta. EVAR would be preferable in such situations, if suitable, and leads to resolution of the inflammation.

We report an IAAA coexistent with a CPK. We could not find an analogous combination in the English literature where the AAA was inflammatory. A case with IAAA has been reported but it refers to CFRE. 7 Until now, a few cases have been reported referring to infrarenal aortic/iliac aneurysm and CPK or CFPK. A latest review included 28 patients. 2 We found additionally 18 patients in 17 case reports and one case series ( Table 1 ). All the 46 patients had non-IAAAs.

**Table 1:** Cases of abdominal aortic/iliac aneurysms and a congenital pelvic kidney or congenital fused pelvic kidney after the latest review 9

References	Year of publication	No of patients (age/sex)	CPK location	Renal ischemia time (min)	Type of aortic repair	Aneurysm type
Loison et al 10	2001	1 (60/M)	Left	50	Open aortobiiliac bypass andreimplantation of the RAs	AAA
Machado et al 1	2015	1 (75/M)	Right	6	Hybrid (Iliac-renal bypass plus EVAR)	AAA
Sarralde et al 11	2015	1 (60/M)	Right	40	Open aortobiiliac bypass andreimplantation of the RA	RCIAA
Banzic et al 12	2015	1 (65/M)	Left	40	Open aortobiiliac bypass andreimplantation of the RAs	Aortoiliac aneurysm
Saito et al 13	2016	1 (54/M)	Solitary	11	Open aortobiiliac bypass andreimplantation of the RA. Graft-SMA bypass	AAA, SMA stenosis
Majumder et al 9	2017	1 (75/M)	Right	≈ 0	EVAR, custom-made fenestrated endograft	AAA
Alves RamosDiniz et al 8	2018	1 (67/M)	Left	30	Open trifurcated graft	AAA
Sakai et al 3	2018	1 (91/M)	Left	≈ 0	EVAR, Bifurcated endograft	AAA
Edwards et al 4	2019	6 (62/M) (71/M) (65/M) (60/M) (79/M) (65/M)	N/A	N/A	2 open Aortic tube graft Aortobifemoral bypass with renal reimplantation 2EVAR Classic EVAR Overlapping aortic cuffs 2hybrid 1AUI-cross-femoral bypass 1Ilio-renal bypass andFEVAR	AAA AAA/RCIAA CIAA Saccular AAA Bilateral CIAAs AAA
Knipe 14	2019	1 (70/M)	Bilateral pelvic	−	No intervention	Small AAA
Ertugay et al 6	2020	1 (63/M)	Solitary	≈ 0	Chimney graft implantation	AAA
Dimic and Sladojevic 15	2020	1 (60/M)	Right	39	Open trifurcated graft	Ruptured AAA
Centofanti et al 5	2021	1 (75/M)	Left	≈ 0	Iliac branch devise	RCIAA

Abbreviations: AAA, abdominal aortic aneurysm;AUI, aorto-uni-iliac; CIAA, common iliac artery aneurysm; CPK, congenital pelvic kidney; EVAR, endovascular aneurysm repair; FEVAR, fenestrated endovascular aneurysm repair; RA, Renal Artery; RCIAA, right common iliac artery aneurysm; SMA, superior mesenteric artery.

Different types of repair have been used in the literature including open, EVAR, and hybrid approaches. Special issues have emerged, as the aberrant arterial anatomy provides unique challenges in preservation of kidney perfusion during operation and minimizing renal tissue damage. Various methods have been employed intraoperatively, which are described briefly in Table 2 . A preoperative careful identification on the CTA of all the ectopic arteries and their orifices is essential to guide treatment.

**Table 2:** Modifications to normal techniques that had been used in the past, to preserve pelvic kidney's viability

Type of operation	Goals of procedure	Techniques
Open	a. Minimization of renal ischemia time	• Shunt: aortorenal, axillofemoral, axillorenal, brachiorenal, double shunt technique • Double aortic clamping • Axillofemoral bypass (temporary, permanent) • Expeditious anastomoses after clamping: “clamp and sew” 7 • Pump oxygenators 4
	b. Preservation of renal tissue during ischemia	• Infusion of special solutions in renal arteries • Cryopreservation of renal tissue (ice slush locally)
	c. Revascularization of ectopic arteries (as much arteries as possible)	• Reimplantation 3 • Bypass 7
EVAR	a. Renal ischemia is not an issue as it occurs only for a few seconds, during the ballooning	
	b. Revascularization of ectopic arteries (as much as possible)	• Chimney technique 6 • Fenestrated endografts 8 • Branched endografts • Standard technique (or with sacrifice of one in case of 2 or 3 ectopic renals, if necessary) 2 3 • AUI andfem-fem bypass 1 3 4 • Iliac branch devise 5
Hybrid 1 7	a. Renal ischemia is not an issue as it occurs for a few minutes only during the renal anastomosis (in a, e.g., iliorenal bypass)	
	b.Revascularization of ectopic arteries (as much as possible)	Same as for EVAR

Abbreviations: AUI, aorto-uni-iliac; EVAR, endovascular aneurysm repair; fem-fem, femoro-femoral bypass.

In our patient, open reconstruction would have been troublesome and risky due to the inflammatory nature of the aneurysm and the retroperitoneal adhesions. The addition of a reperfusion technique like an axillorenal shunt would have rendered the operation more complex. 2 The reimplantation of the proximal renal artery with a Carrel patch posed a concern due to the thick inflammatory orifice. Under these circumstances, a renal bypass might be preferable. 8 Renal ischemic time is another important issue and a cryopreservation method would be necessary.

We proposed to the patient to undergo a hybrid repair: an open iliorenal bypass with a saphenous vein graft aiming to revascularize the proximal renal artery, followed by a standard EVAR. 1 Renal ischemic time would have been restricted only during the renal anastomosis and the ballooning. Nevertheless, our patient declined this option.

A chimney or branched/fenestrated graft approach was not chosen due to the small size of the proximal renal artery and the long length required. 6 9

After obtaining the patient's consent, we proceeded with a standard EVAR at the cost of sacrificing one out of two aberrant renal arteries. There is one report in the literature with the same treatment strategy and a second one where sacrifice of one out of three aberrant renal arteries was specified. 3 4 In both cases, the AAA was noninflammatory. This option is acceptable in patients with a second normally located kidney, but sacrifice of ectopic arteries should rather be avoided in solitary kidneys. 1

In conclusion, we believe that standard EVAR provides a high-success, low-risk option to deal with AAA repair in case of a concomitant CPK. Sacrifice of one ectopic artery is justified, when other options are risky, complex, or not acceptable to the patient.
